# The Comparison of Nailfold Capillaroscopy between Juvenile Systemic Lupus Erythematosus and Healthy Controls: Correlation with Laboratory and Clinical Parameters

**DOI:** 10.1155/2020/7631958

**Published:** 2020-04-27

**Authors:** Seyed-Reza Raeeskarami, Navid Namazi, Raheleh Assari, Seyed-Reza Najafizadeh, Zohreh Hassannejad, Vahid Ziaee

**Affiliations:** ^1^Department of Pediatrics, Tehran University of Medical Sciences, Tehran, Iran; ^2^Pediatric Rheumatology Research Group, Rheumatology Research Center, Tehran University of Medical Sciences, Tehran, Iran; ^3^Children's Medical Center, Pediatrics Center of Excellence, Tehran, Iran; ^4^Rheumatology Research Center, Valiasr Hospital, Tehran University of Medical Sciences, Tehran, Iran

## Abstract

**Background:**

Nailfold capillaroscopy is a noninvasive technique to recognize peripheral microangiopathy, which is an important feature in systemic lupus erythematosus (SLE). The aims of the present study were to investigate the prevalence of nailfold capillaroscopy (NFC) changes in juvenile systemic lupus erythematosus (JSLE), find out patterns of these changes, and correlate findings with clinical and laboratory parameters.

**Methods:**

Forty-nine patients with SLE, all meeting the 1997 revised ACR criteria for SLE classification, and 30 healthy controls were included. A digital video camera was used to capture nailfold capillary images. Computerized image processing was used for analysis.

**Results:**

Different abnormal NFC changes were observed with abnormal morphology, the increased diameter and abnormal loop densities in 55.1%, 93.9%, and 26.5% of the patients, respectively. No statistically significant differences were depicted between capillaroscopy with age, gender, autoantibodies (APLs, anti-ds DNA), antiphospholipid antibody syndrome, thrombotic angiopathy, renal function tests (Bun, Cr), and abnormal urine analysis. However, a significant correlation was found between the branched pattern and the CNS involvement group (*P* value <0.03).

**Conclusions:**

Different abnormal NFC changes are quite common among patients with SLE, and nailfold capillaroscopy is an effective method to monitor such changes. Treatment strategies may change in the branched pattern of nailfold capillaroscopy due to CNS involvement.

## 1. Introduction

Juvenile systemic lupus erythematosus (JSLE) is a rare, multisystem and potentially autoimmune disorder with significant morbidity. Clinical manifestations in lupus may be more prevalent and severe in children [1]. Vascular pathologies in lupus including thrombotic events, microinfarcts, vasculitis, and perivascular inflammation could be responsible for heterogenous clinical manifestations [2].

Nailfold capillaroscopy (NFC) is a highly sensitive, simple, and noninvasive imaging technique used in morphological analysis of capillaries.

To our knowledge, no study was undertaken to investigate the prevalence of NFC patterns in patients with JSLE in comparison with age-matched healthy controls. In this study, we attempted to examine and compare NFC pattern prevalence in JSLE patients and age-matched healthy individuals. In addition, we intended to assess whether the presence of nailfold capillary anomalies would be associated with clinical and laboratory parameters.

## 2. Patients and Methods

Forty-nine pediatric SLE patients, meeting the updated 1982 ACR revised criteria [3], and 30 healthy children were included in the study. Informed consent was obtained from parents of all the children. Demographic characteristics, clinical findings, and laboratory tests including autoantibodies (APLs, anti-ds DNA), renal function tests (Bun, Cr), and urine analysis were documented for the patients.

Neurologic involvement in SLE has various symptoms and signs, including seizure, cerebrovascular disease, loss of consciousness, guillain barre, transverse myelitis, and neuropathies. Headache, mild cognitive disorder, and mood disorder were excluded from the central and peripheral nervous system symptoms and signs.

After sitting at the temperature of 20–22°C for 15–20 minutes, a drop of immersion oil was applied to the nailfold before capillaroscopy. Direct capillaroscopy was performed using the digital video-capillaroscope (optipix-optilia OP 120 021, Sweden) OptiPix capillaroscopy software equipped with a ×200 fiber optic illumination. Nail folds of all fingers were examined in the patients and controls, and two high-quality images (1 × 1 mm) were obtained.

The capillary density, width, morphology, and arrangement were assessed by two blind observers using the morphology, diameter, architecture, and density approach [4].

**Figure 1:**
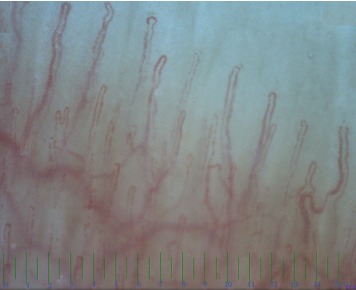
Normal morphology, structure, diameter and density are seen in nail fold capillaroscopy in a normal 8 years old girl.

In the definition of tortuosity, hairpin and curly changes are interpreted as normal alternations, while other variations are involved as tortuous (serpentine) changes [4].  Figure 1 shows the normal pattern of a nail fold capillaroscopy in a healthy child.

The increased diameter was divided into irregular and regular patterns. In the irregular pattern, a segment of the capillary was wider than the other segments, whereas, in the regular pattern, the entire vessel was wide.

Statistical significance for various associations was calculated using *χ*2 analyses or Fisher's exact test, as appropriate. Moreover, the linear regression method was used to analyze the relationship between overall capillaroscopic patterns and disease characteristics. *P* < 0.05 was considered as significant.

## 3. Results

A total of 49 SLE pediatric patients (28.6% male) and 30 controls were evaluated. The mean age of the patients was 11.55 ± 3.84 (6–17) years. The control group was age and sex-matched and had a mean age of 12.33 (±3.84) years. The demographic and clinical characteristics of the patients are shown in Table 1.

The JSLE patients had 55.1% (22.4% branched and 32.7% tortuosity) abnormal morphology, 93.9% (85.7% irregular and 8.2% regular) enlarged diameter, 8.2% disorganized architecture, and 26.5% reduced density.

**Figure 2:**
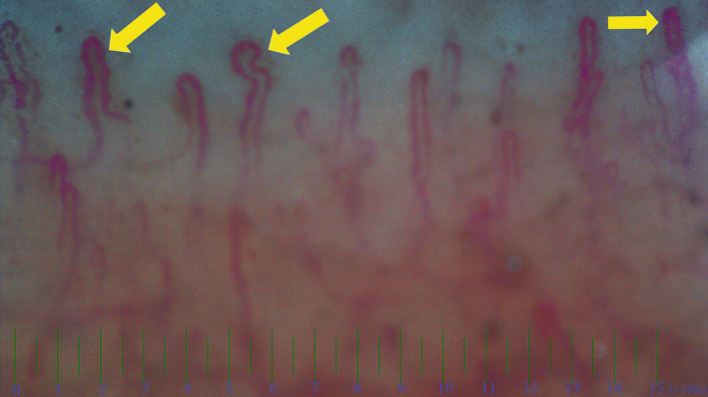
Nail fold capillaroscopic changes in a 10 years old boy with juvenile systemic lupus erythematosus and nephritis. Arthritis following nephritis and hematologic involvement was presented at 5 years old as the first manifestations. The increased diameter in the irregular pattern was demonstrated in the nail fold capillaroscopy of this patient.

Abnormal morphology, enlarged diameter, and reduced density were significantly associated with SLE diagnosis (*P* < 0.05) (Table 2).  Figure 2 shows the irregular pattern and increased diameter in the nail fold capillaroscopy of one of the SLE patients.

**Figure 3:**
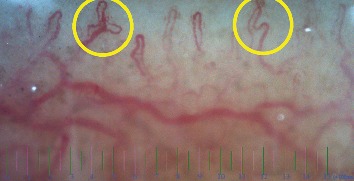
Nail fold capillaroscopic changes in an 8 years old girl with juvenile systemic lupus erythematosus and C1q complement. Central nervous system involvement (motor developmental delay) and skin lesions were presented at 2 years old as the first manifestations. Loss of density (<7/mm) and branch pattern were demonstrated in the nail fold capillaroscopy of this patient.

No statistically significant correlation was found between capillaroscopy patterns with age, gender, autoantibodies (APLs, anti-ds DNA), antiphospholipid antibody syndrome, thrombotic angiopathy, renal function tests (Bun, Cr), and abnormal urine analysis. However, a significant correlation was found between the branched pattern and the CNS involvement group (*P* value = 0.03). The loss of density and the branched pattern were detected in the nail fold capillaroscopy of a SLE patient with C1q deficiency and CNS involvement, as demonstrated in Figure 3.

## 4. Discussion

SLE is a heterogeneous disease with various vascular involvements. Changes of capillaroscopy depend on the active phase of the disease. Thus, no specific pattern has been defined for SLE [5]. However, the loss of capillaries and specific SSC patterns were defined in systemic sclerosis due to vascular obliteration [6]. SLE patterns for adults are described in the literature [7, 8]. In juvenile systemic lupus erythematosus, few data are available about the capillaroscopy pattern [9–12]. Capillary changes in pediatrics are age-dependent, especially for capillary density and capillary dimensions [13]. No previous studies compared capillaroscopic changes with healthy controls [5]. As we know, this was the first study that compared capillaroscopy changes in juvenile SLE with healthy age-matched controls.

In this study, tortuosity morphology was significantly higher in the SLE patients than in the controls. Although some studies have described tortuosity as a normal variation [13, 14], others have defined tortuosity as an SLE pattern [7–9]. Tortuosity is age-related and thus increases during childhood [13]. Nevertheless, this morphology was not observed in the control group in the current study. The velocity of tortuous progress may be more in JSLE patients than in healthy controls, although this abnormality is nonspecific changes, like adulthood. Therefore, the presence of tortuosity in JSLE can be an important sign.

The mean diameter of the capillary and irregular pattern is related to SLE disease [5, 7–9]. In the present study, disarrangement and the disorganized architecture were not significantly high in the SLE patients, unlike other literature [5, 7–9]. Disorganized architecture changes in juvenile lupus patients may be a prolonged process, which may even last up to adulthood.

In the present study, a density of less than 7 mm was observed in the SLE patients compared to the controls. Other studies demonstrated no significant correlation between age and capillary length density in JSLE [9–11]. However, this correlation has been observed during adulthood [5, 8]. In children older than 10 years, the nailfold capillary density reaches density in adulthood [13]. The mean age of the SLE patients and its comparison with that of the age-matched healthy controls in the present study may demonstrate the importance of density (less than 7 mm) in JSLE.

In the present study, no correlation was found between antiphospholipid antibodies with anti-ds DNA and capillary alterations. Although some studies considered capillaroscopic changes as a screen test for the antiphospholipid syndrome in rheumatic disease [15, 16], others found a correlation between capillaroscopic changes and the antiphospholipid syndrome [17]. Further longitudinal evaluation should be considered in this regard.

In addition, no correlation was found between renal function tests and overall capillaroscopic patterns, which is similar to the findings of previous studies [9, 14, 17, 18].

The branched capillary pattern is one of the specific abnormalities in the SSC pattern. Local tissue anoxia resulted in autoregulative responses; thus, neovascularization occurred for compensation purposes and the branched pattern was observed. As a result, even one branched pattern could be important [19].

In this study, the branched pattern of capillaroscopy and the CNS involvement were significantly correlated (*P* value <0.03). There are limited studies about capillaroscopy changes and central nervous system findings in SLE or other rheumatologic diseases, and no statistically significant correlation has been found between them [9, 14, 17, 18].

The role of multiple pathologic mechanisms has been confirmed in SLE manifestations such as neuropsychiatric disorders. Clinical CNS manifestations such as headache, anxiety, mild cognitive disorders, and mood disorders do not reflect SLE activity in CNS [20]. Thus, they are excluded from the CNS activity group with seizure, cerebrovascular disease, loss of consciousness, guillain barre, transverse myelitis, neuropathies, and others [20, 21]. No correlation has been found between antiphospholipid antibodies and branched patterns. Antiphospholipid antibodies have a significant role in hypercoagulative state or a direct effect on neurons in neuropsychiatric manifestations in SLE. The focal CNS manifestation may be due to vascular occlusions such as thrombosis, vascular ischemia, and CNS vasculitis. Although the main causes of CNS involvement have not been defined, the vascular integrity damage (antiplatelet antibody, stress, occlusion, inflammation, and others) causes blood-brain barrier destruction and increased BBB permeability. Subsequently, local tissue anoxia results in reperfusion [22]. The same pattern may cause a branched pattern in the microvascular loop in the capillaroscopy of JSLE patients.

In conclusion, significant differences were observed in terms of tortuosity, the branched pattern, the reduced density, and the enlarged vessel diameter in juvenile systemic lupus erythematosus compared to healthy controls. Moreover, a significant correlation was found between the branched pattern and the CNS involvement. Thus, the branched pattern in the capillaroscopy of SLE patients may change treatment strategies in CNS activity. However, no significant correlation was found between the other clinical and laboratory parameters with capillaroscopic alterations. Further extensive studies should be considered to confirm the findings of the present study.

## Figures and Tables

**Table 1:** Main demographic and clinical features of the patients with systemic lupus erythematosus (*n* = 49).

Demographic and clinical variables	*N* (%)	Laboratory variables	*N* (%)
Male	14 (28.6)	Anti-double strand DNA	15 (30.6)
Female	35 (71.4)	BUN increased	3 (6)
New case	5 (10.2)	Cr increased	6 (12.2)
Chorea	1 (2)	U/A abnormality∗	44 (89.7)
New or worsening headache	32 (65.3)	Anti-beta glycoprotein 1 (IgM)	0
Antiphospholipid antibody syndrome	4 (8)	Anti beta2 glycoprotein 1 (IgG)	1 (2)
Seizure	5 (10.2)	Anticardiolipin ab (IgM)	2 (4)
Loss of consciousness	4 (8)	Lupus anticoagulant	3 (6)
Behavioral changes	10 (20.4)	Vascular thrombosis	6 (12.2)

∗U/A (urinalysis) abnormality: Pr > +3 or casts.

**Table 2:** The number, percentage, and *P* value of capillaroscopic changes between juvenile systemic lupus erythematosus and healthy controls.

	JSLE (49) *N* (%)	Control (30) *N* (%)	*P* value
Abnormal morphology	27 (55.1%)	1 (3.3%)	<0.001
Tortuosity	16 (32.7%)	0	<0.001
Branched	11 (22.4%)	1 (3.3%)	<0.025
Increased diameter	46 (93.9%)	1 (3.3%)	<0.001
Irregular	42 (85.7%)	1 (3.3%)	<0.001
Regular	4 (8.2%)	0	0.141
Irregular architecture	4 (8.2%)	0	0.141
Density (less than 7)	13 (26.5%)	0	0.001

JSLE: juvenile systemic lupus erythematosus.

## Data Availability

The data used to support the findings of this study are available from the corresponding author upon request.
